# A patient with cystic duct remnant calculus treated by laparoscopic surgery combined with near-infrared fluorescence cholangiography

**DOI:** 10.1186/s40792-020-00909-7

**Published:** 2020-06-23

**Authors:** Shinichi Matsudaira, Tsuyoshi Fukumoto, Akinaga Yarita, Joji Hamada, Masayuki Hisada, Junichi Fukushima, Nobuaki Kawarabayashi

**Affiliations:** 1 Department of Digestive and General Surgery, Gyoda General Hospital, 376, Motida, Gyoda-shi, Saitama, 361-1156 Japan; 2 Department of Emergency and General Practice, Gyoda General Hospital, Saitama, Japan; 3 Department of Diagnostic Pathology, Gyoda General Hospital, Saitama, Japan

**Keywords:** Remnant cystic duct, Near-infrared fluorescence, Post-cholecystectomy syndrome

## Abstract

**Background:**

The recurrence of symptoms present before cholecystectomy may be caused by a cystic duct remnant. The resolution of cystic duct remnant syndrome may require surgical resection, but identification of the duct remnant during laparoscopic surgery may be difficult because of adhesions following the previous procedure. Open surgery, which is more invasive than laparoscopic surgery, is frequently chosen to avoid bile duct injury.

**Case presentation:**

The patient was a 24-year-old woman with previous laparoscopic cholecystectomy for chronic cholecystitis and repeated attacks of biliary colic. The postoperative course was uneventful, but computed tomography revealed a remnant cystic duct calculus. Ten months after surgery, the patient returned to our department for right hypochondriac pain. Laparoscopic remnant cystic duct resection was performed with intraoperative near-infrared (NIR) fluorescence cholangiography to visualize the common bile duct and remnant cystic duct. The postoperative course was uneventful and the patient was discharged on day 3 after surgery. At the 6-month follow-up, she had no recurrence of pain.

**Conclusion:**

Laparoscopic surgery with NIR cholangiography is a safe and effective alternative for the removal of a cystic duct remnant calculus after cholecystectomy.

## Background

Post-cholecystectomy syndrome (PCS) is a recurrence of upper abdominal and right hypochondriac pain, jaundice, dyspepsia, gastrointestinal disorders, and vomiting experienced before cholecystectomy [1]. PCS may be caused by a remnant cystic duct. Resolution of cystic duct remnant syndrome (CDRS) may require surgery [2], but intraoperative laparoscopic identification of a remnant cystic duct can be difficult because of adhesions that formed after the previous surgery. Even though it is more invasive, open surgery may be chosen in such cases to avoid bile duct injury. Intraoperative near-infrared (NIR) fluorescence cholangiography with intravenous injection of indocyanine green (ICG) can be used to visualize the cystic duct and common biliary duct and is an alternative to conventional cholangiography [3]. This patient with a cystic duct remnant calculus was treated by laparoscopic surgery combined with NIR fluorescence cholangiography. A brief review of the relevant literature is included.

## Case presentation

**Fig. 1:**
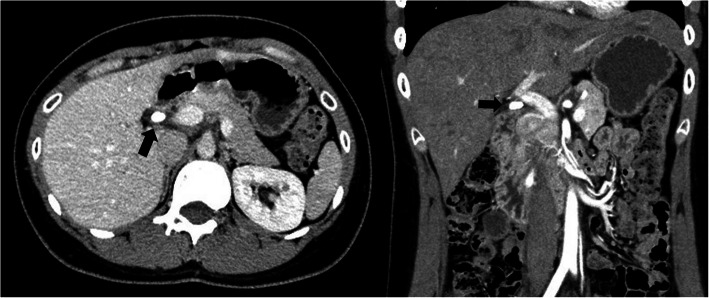
CT showing a cystic duct remnant calculus (arrow)

**Fig. 2:**
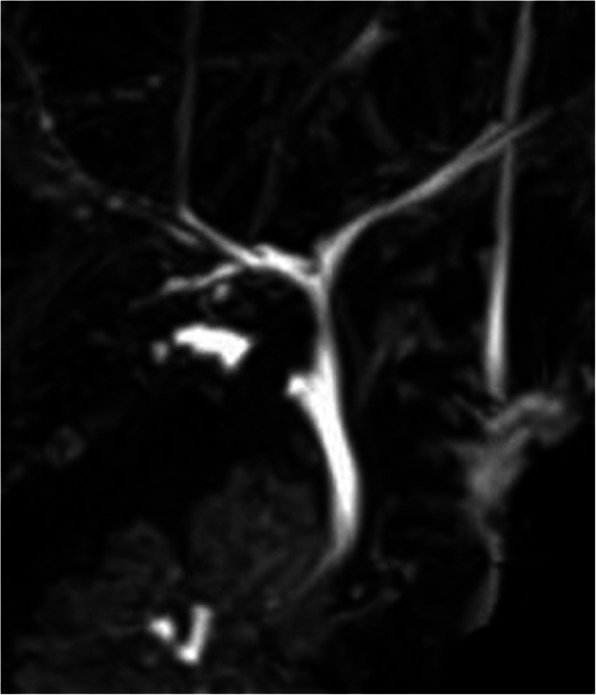
MRCP showing the remnant cystic duct of 2.0 cm in length and a calculus

**Fig. 3:**
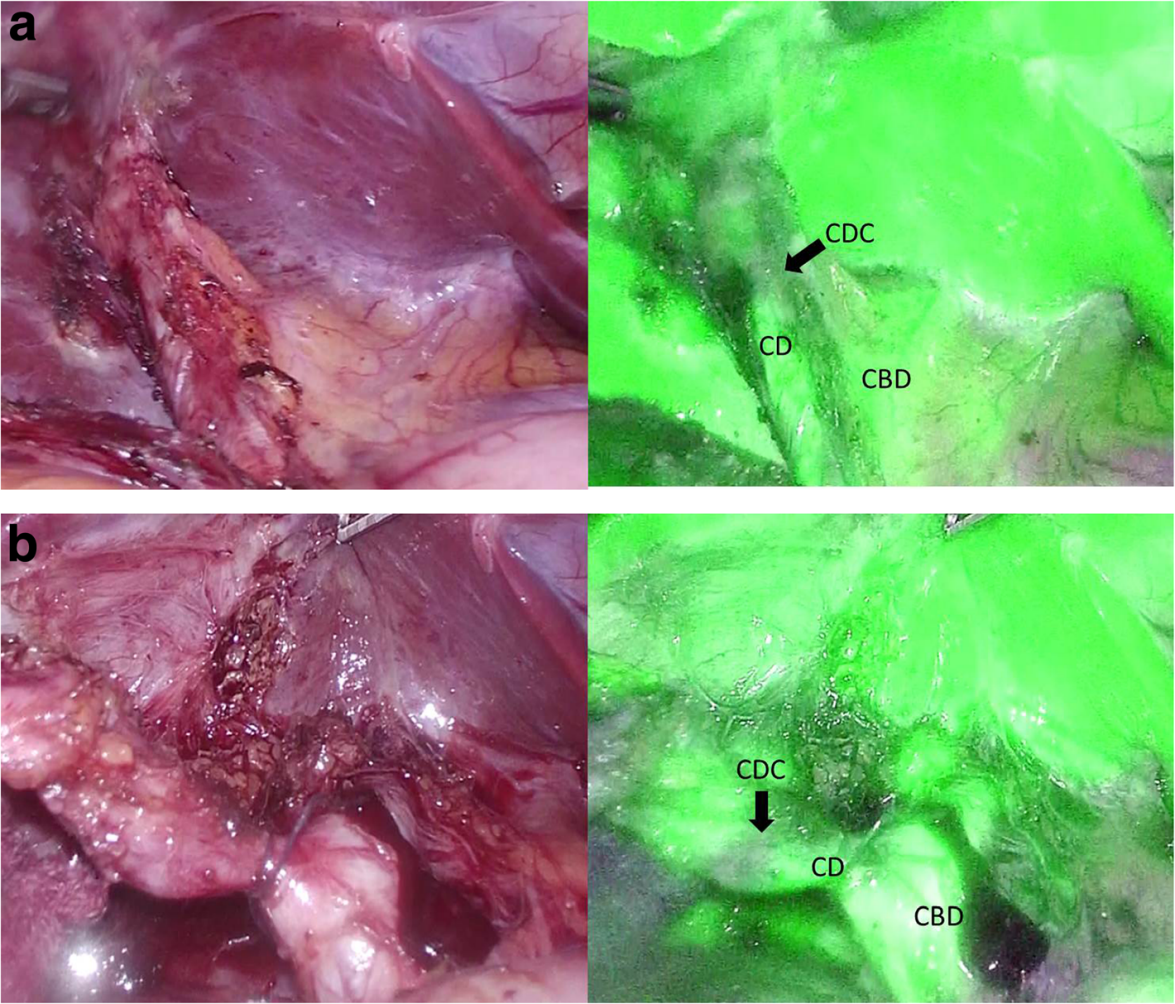
**a** Color and fluorescence image before dissection of adhesion around the remnant cystic duct. **b** Color and fluorescence image after dissection of adhesion around the remnant cystic duct. CD, cystic duct; CBD, common bile duct; CDC, cystic duct calculus

A 24-year-old woman with chronic cholecystitis and episodes of biliary colic underwent laparoscopic cholecystectomy at our hospital. Although preoperative computed tomography (CT) revealed a cystic duct calculus, the complete removal of the calculus could not be confirmed considering bile duct injury because there were moderate chronic inflammations around the cystic duct. The postoperative course was uneventful and without pain. Ten months later, the patient visited the outpatient clinic because of hypochondriac pain. Laboratory results showed normal liver function, and inflammation and jaundice were absent. CT revealed a cystic duct remnant calculus at the same site as before (Fig. 1). Magnetic resonance cholangiopancreatography revealed a 2 cm calculus in the remnant cystic duct (Fig. 2) and a normal pancreaticobiliary junction. Esophagogastroduodenoscopy revealed no abnormalities. The findings were consistent with pain caused by the cystic duct remnant calculus. The patient wished to avoid a surgical scar and consented to laparoscopic resection, which was performed with NIR cholangiography to reduce the risk of bile duct injury. At the beginning of the procedure, 1 ml ICG (2.5 mg/ml Diagnogreen; Daiichi Sankyo, Tokyo, Japan) was injected intravenously. Four-port laparoscopy was performed via the scars remaining from the previous procedure. A 12-mm camera port was placed at the umbilical area by the open method, a 12-mm port was placed in the epigastric area, a 5-mm port was placed in the right hypochondriac area, and a 5-mm port was placed in the right lateral abdomen. Adhesions at the gallbladder bed and the lateral and ventral hepatoduodenal ligament were dissected. NIR fluorescence cholangiography was started using the fluorescent imaging system and a CLV-S200-IR/WAIR130A xenon light source (OLYMPUS, Tokyo, Japan) about 40 min after ICG injection. NIR fluorescence cholangiography visualized the common bile duct, remnant cystic duct, and cystic duct remnant calculus (Fig. 3a, b). An adhesion around the remnant cystic duct was identified by fluorescence imaging and was dissected. The remnant cystic duct stump was ligated by double end-loop sutures (Endoloop PDS II; Ethicon Inc., Cincinnati, OH, USA). The remnant cystic duct was resected. The procedure ended without intraoperative complications after 1 h and 17 min and a blood loss of 10 ml. Macroscopically, the resected specimen was 1.8 cm and the calculus was 1.0 cm long. The postoperative course was uneventful, the pain resolved soon after surgery, and the patient was discharged on day 3 after surgery. At the 6-month follow-up, there had been no recurrence of pain.

## Discussion

PCS is characterized by the recurrence of symptoms experienced before cholecystectomy [1]. In CDRS, the symptoms are caused by a cystic duct remnant up to 1 cm long [4]. The presence of calculi may produce the symptoms. Postoperative symptoms associated with cystic duct remnants were first reported by Florcken in 1912 [5], and Rozsos et al. reported an incidence of PCS as high as 16% [6]. Resolution of symptoms may require surgical resection of the remnant cystic duct, but adhesions that formed after the initial surgery can make intraoperative identification of the remnant difficult. Palanivelu et al. reported that successful laparoscopic resection of cystic duct remnant calculi was dependent on the experience of the surgeon [7], but NIR fluorescence cholangiography has helped to reduce the dependence on experience. In NIR fluorescence cholangiography, ICG excreted into the bile and protein-bound ICG emit fluorescence with a peak wavelength of approximately 830 nm when illuminated with near-infrared light [8]. Ishizawa et al. first described laparoscopic cholecystectomy with NIR fluorescence cholangiography following intravenous injection of ICG [9]. Currently, an ongoing randomized controlled trial has been conducted to confirm the efficacy of NIR fluorescence cholangiography during cholecystectomy [10]. ICG is excreted into bile within minutes, but it should be administered at least 15 min in advance to minimize background fluorescence [11]. Kono et al. described that the optimal time of injection is approximately 90 min before observation [12]. ICG was administered to our patient when the procedure started because of the estimated time (at least more than 60 min) required to dissect adhesions of the hepatoduodenal ligament. NIR fluorescence cholangiography began about 40 min after ICG injection, which may have resulted in a relatively high background signal. As a result, ICG should have been injected earlier than the time when the procedure was started.

Previous systematic reviews have described the potential advantages of fluorescence cholangiography over conventional cholangiography during laparoscopic cholecystectomy [13]. In addition, resection of a remnant cystic duct is not easy to perform by conventional cholangiography. An endoscopic nasobiliary drainage tube must be placed before surgery because intraoperative insertion of a cannula to inject contrast material for biliary tree imaging is not possible. That increases patient discomfort and may cause a number of complications. Preoperative intravenous injection of ICG is an advantage of NIR fluorescence cholangiography. Labrinus et al. recently described the use of NIR fluorescence imaging during robot-assisted resection of remnant cystic ducts [14]. They recommended NIR fluorescence imaging with ICG during difficult robotic surgical procedures of the biliary tract, such as resection of remnant cystic ducts and gallbladder surgery when inflammation of surrounding tissue is expected. Robotic surgery may make it possible to perform procedures more delicate than laparoscopic surgery, but resection of the remnant cystic duct by laparoscopic surgery can be safely performed with NIR fluorescence imaging.

## Conclusion

This patient was safely and successfully treated for a cystic duct remnant calculus by relatively noninvasive laparoscopic surgery in combination with NIR fluorescence cholangiography and ICG.

## Data Availability

All datasets presented in the main paper are available whenever possible.

## Abbreviations

NIR: Near-infrared
PCS: Post-cholecystectomy syndrome
CDRS: Cystic duct remnant syndrome
ICG: Indocyanine green
CT: Computed tomography
